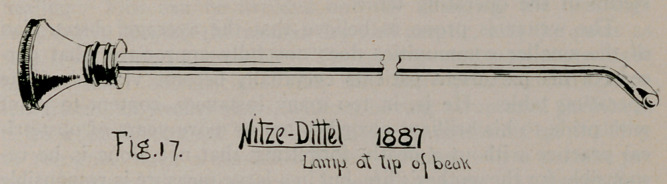# Originality and Priority in Modern Cystoscopes

**Published:** 1908-08

**Authors:** Bransford Lewis

**Affiliations:** Saint Louis


### BUFFALO MEDICAL JOURNAL.

Vol. Lxiv. AUGUST, 1908. No. 1

### ORIGINAL COMMUNICATIONS.

### Originality and Priority in Modern Cystoscopes.

**A REPLY TO DR. F. TILDEN BROWN.**

By BRANSFORD LEWIS, M. D., SAINT LOUIS.

IN the *Medical Record* of February 29, 1908, and in the *New York Medical Journal* of April 11, 1908, Dr. F. Tilden Brown, of New York, is quoted as presenting a paper on Ureteral Catheterisation, before the meeting of the Medical Association of the Greater City of New York, held January 20, 1908. In this paper the essayist makes certain well-defined claims to originality and priority with respect to his instruments, and also claims that his originality has been trenched upon. The allegations are so worded as to leave no doubt that I am the one accused, and also leave no doubt as to the necessity of a reply, which is herewith presented.

In order that Dr. Brown's position may be correctly stated, his own words shall be used, as found in the publications mentioned, as well as in expressions of his in other contributions. Quotations from the two journals (practically the same in both) follow:

"Dr. Brown's remarks were directed more particularly to instruments and methods with which he himself had to do, and were supplemented by wall drawings and photographs to demonstrate the various developmental forms, since 1900, of his original 'composite cystoscope,' the identical instrument which a St. Louis cystoscopist had recently appropriated, and had been presenting as his own 'universal cystoscope.' This composite cystoscope, made by the Wappler Co., of New York, was a vastly more useful instrument than its immediate predecessor, the author's double-catheter direct vision cystoscope, which Leiter, of Vienna, made for him in 1898, and which was the first telescopic cystoscope, of any form, to provide for two catheters and effect synchronous catheterism of the ureters. But this Vienna instrument had no reserve channels for irrigation. It was with the end in view of finding a way to add such irrigation channels to the already practicable double-catheter direct vision cystoscope, while, at the same time, not increasing the circumference of the shaft beyond

24 French, that the Brown-Wappler efforts were first directed, in 1900. With what initial success these efforts had met and what constantly added improvements he expected, the present presentation was intended to fully demonstrate. It should be here added that the speaker's first double-catheter instrument was but a modification of the then existing Brenner single cystoscope; whereas his subsequent instruments involved a wholly novel departure from all the preëxisting types. The first essential change lay in getting rid of the old-time terminal window at the vesical end of the sheath, which was followed by the use of different kinds of interchangeable telescopic tubes for the same common sheath. Up to the present time its development on this basis had resulted in giving us at least three complete cystoscopes for different purposes, adjustable in one sheath. Still another tube, of paramount value, to go with this common sheath, was all but completed, in the shape of an indirect vision, double-catheter telescope which would be welcome to those who preferred this method of ureter catheterism to the direct one, and which here in

Fig. 1. Brenner

America had been gaining constantly in favor since the first introduction of the composite cystoscope."

In another contribution[1] Dr. Brown expresses his claim to priority regarding the double catheter tubes in the following terms:

"The speaker may be pardoned if, influenced by an experience of this kind and from a source so little expected, he asks now to place on record the fact that his was the first cystoscope devised for and successfully used to catheterise both ureters at the same time."

From these several quotations from Dr. Brown I believe we can fairly assume that his claims and complaints are embodied in the following three propositions:

1. That in modifying Brenner's (Fig. 1) single catheterising cystoscope in 1899[2], by supplying it with two catheterising channels instead of the one it already possessed, he promulgated, as he says, his "double catheterising, direct vision cystoscope,

1. Med. and Surg. Reports of Bellevue Hospital, Vol. I., January, 1905, p. 368.

2. So dated by Dr. Brown in his first publication of the instrument in the Annals of Surgery, 1899, p. 661, although he would now lead us to infer that it was 1898 (see first quotation. The composite is attributed to 1901 (instead of 1900) by the Wappler Electric Controller Co., in the following extract from a letter to Dr. Brown: "On April 30, 1901, we made for you [Brown] your first composite cystoscope.—Wapp. E. C. Co."

which was the first telescopic cystoscope, of any form, to provide for two catheters and effect synchronous catheterisation of the ureters."

2. That his composite cystoscope of 1901" involved a wholly novel departure from all the preëxisting types."

3. That in planning my universal cystoscope, presented in 1906, I copied his composite instrument.

Before answering these propositions directly, I must advert to some facts of cystoscopic history. Modern cystoscopes are divisible into two groups or classes: (a) those of fixed-lens systems, in which the lenses are inseparably connected with the shaft of the instrument, the whole being permanently assembled into one piece; (b) those in which the cystoscope is separable into

Fig. 2. Boisseau du Rocher (1889)

two principal parts—namely, a sheath, and a telescope or ocular part.

With one exception, to be mentioned later, all of the cystoscopes submitted by the father of modern cystoscopy, Nitze, were of the first group; the departure from this plan, as exhibited in the sheath-and-telescope group, was devised and developed by Boisseau du Rocher, of France, and the full credit for originating it is due to him. His first instrument, devised and used by operators of Paris as early as 1889, was published in the *Annales des maladies des organes genito-urinaires,* 1890, pp. 65-93. It embodied the following remarkable features, as shown by the description and illustrations (Fig 2) of the author, as well as by numerous writings of others of the succeeding fifteen years—namely, a sheath, bearing a removable electric incandescent lamp in the chamber of its beak, illuminating the field on the convexity of the instrument; in its lower wall two small channels for the definite purpose of conducting two ureteral catheters, and for effecting change of the fluid in the bladder, or irrigation. The sheath was supplied with an obturator, which facilitated the introduction of the cystoscope into the bladder, after which the obturator was replaced by the telescope or ocular apparatus, that gave the direct view within the bladder.

Describing different features of this sheath, (ibid. p. 73) and proving the definite purpose of double synchronous catheterisation fulfilled by the tubes, the author says: "On the lower side of the straight sheath run two parallel tubes with internal caliber of No. 6, French scale. These tubes open immediately behind the window, o. At the other (ocular) extremity they terminate at the cocks r r; and contain obturators m m to facili-

Fig. 3.

Boisseau du Rocher – (1898)

tate the passage of the instrument over the urethral mucous membrane. These small tubes have two different purposes: they serve, in the first place, as conductors for the ureteral catheters. Nothing is simpler, having observed the orifice of the ureters, with the catheter in situ, than to guide the latter and engage it in the ureter. By pushing it from the outside, one may insert it to the depth desired. Finally, these small catheters being controlled and independent, every facility is furnished for catheterising the two ureters without the necessity of withdrawing the instrument or disengaging the first catheter.

"A second purpose, also very important, fulfilled by the two little tubes, is that of irrigation of the bladder. They constitute, in reality, a double-current catheter, by means of which one may effect irrigation, if not complete and rapid, at least sufficient to maintain the transparency of the liquid."

This model of Boisseau's instrument furnished the field of view and of manipulation on the convex side. In order to obtain a view on the concavity (the right-angle view) a second sheath was constructed with the lamp exposed by a fenestrum on the concavity (Fig. 2) with another fenestrum at o, opposite to which the prism of the telescope came when it was introduced into the sheath. So that these two instruments of 1889 furnished, together, all of the objects furnished by Brown's first cystoscope of ten years later—namely, by the sheath-and-telescope plan: (1) direct view for inspection ; (2) indirect view for inspection; (3) irrigation of the bladder, either through the two catheter tubes during manipulation, or through the sheath when obturator and telescopes were not in place; and finally, (4) double-bar-

Fig 4 Güterbock - 1895

Sheath Fig. 5. Fenwick relled catheter channels for synchronous catheterisation of both ureters at one sitting.

The faithfulness with which Dr. Brown followed the pioneer example here set is an eloquent testimonial of his appreciation of the work of Boisseau,—more eloquent, it must be said, than any written acknowledgment on the subject that I have been able to discover. On the contrary, in his writings we find him claiming, as late as February of this year, the full and complete credit of having introduced "the first telescopic cystoscope, of any form, to provide for two catheters and effect synchronous catheterisation of the ureters." The catheter-tubes of Boisseau were carried within the wall of the sheath; those of Brown in the wall of the telescope. This involved a difference in the location, merely, of the same features.

That this instrument of Boisseau received wide recognition, and that the definite purposes above mentioned were realised by the profession, is indicated in almost every contribution which discusses cystoscopy in a broad way, written in the next following fifteen years. In speaking of his experience in catheterising ureters with Boisseau's instrument *Annales des Maladies des organes genito-urinaires,* 1889, p. 626), Poirier mentions that the difficulties that the cystoscope meets with in the case of tumors of the bladder do not exist in applying it to catheterisation of the ureters; that this operation is very easy. After only a small experience, the operator easily found the orifice of the ureters and

Fig. 6.

Albarran – (1897)

with the small catheter conducted by the particular canal included in the cystoscope, easily penetrated the ureter. He mentions two instances in which both he and his assistant had successfully conducted the manipulations on living individuals in the previous month. His final conclusion was that surgery was henceforth in possession of an easy and practical means of obtaining the separation of the secretions from the two kidneys.

Burckhardt, in *Zuelzer's Handbuch der Harn und Sexualorgane,* Vol. III., p. 158, describes Boisseau's instrument at length, and speaks of the small channels serving for the purpose of introducing the ureter catheters into the ureters.

In 1898 *Annales des maladies des organes genitourinaires.* p. 485), Boisseau submitted an additional model of his sheath-and-telescope plan, in which, by double fenestration of the beak, and multiple ocular telescopes, views were afforded on both concavity and convexity of the same instrument (Fig. 3). One year after this presentation of Boisseau's third model, the first one of Brown appeared,—a double-barrelled modification of Brenner's, as he terms it (*Annals of Surgery,* 1899), and in the illustration of the paper, then presented, designates it with the date, 1899, (Fig. 10).

Thus we have in Boisseau's instruments of 1889 and 1898 all of the essential features of Brown's of 1899 and 1901; but especially do we find in them the definite and complete negation of his claim, repeatedly made, that his "double-catheter direct vision cystoscope was the first telescopic cystoscope, of any form, to provide for two catheters and effect synchronous catheterisation of the ureters."

Nitze-

Fig. 7. Evacuation cystoscope. (1897)

Dr. Brown's claim to this effect is all the more surprising when we observe the evidence of his acquaintance with this feature of Boisseau's instrument, in the allusion he makes to it in his contribution of 1899 to the *Annals of Surgery.* In speaking of the efforts that had been made to provide the Nitze-Leiter cystoscope with a separate channel for the conveyance of a ureteral catheter, with the belief that such a contrivance could be as successfully used in men as in women, Dr. Brown says: "The first instrument of this class was that of Dr. Brenner (1887), and Boisseau du Rocher's shortly after."

But even more significant and surprising than this is the remark of Dr. Brown, following immediately afterward, in the same paper, that "They (Brenner and Boisseau) utilised the Nitze-Leiter cystoscope which had its window opposite the eyepiece, and a catheter outlet just below the window." "A catheter outlet," forsooth! That is a scant concession to make to Boisseau's instrument, under the circumstances. It looks questionable, to say the least, in connection with the presentation of the so-called "first telescopic cystoscope of any form to provide for two catheters and effect synchronous catheterisation of the ureters." The only alternative would have been to come out frankly and refer to the two catheter outlets of that instrument.

In this connection, I have a confession of my own to make; that previous to my late researches into this subject, in common with many others, I have been accepting Dr. Brown's dictum about this double-barrelled catheter feature, and have been in the habit of ascribing to him the credit of originating it, as applied to Brenner's instrument. But I can conscientiously do that

Fig. 8. Koch-Preston-1898 (air)

no longer. Even aside from Boisseau having anticipated him by ten years in presenting the double-barrel feature, his statement does injustice to Casper's cystoscope, which, first introduced in 1894, was improved in 1898 in a manner that presented a means of effecting double synchronous catheterisation by the sliding removable cover to the catheter channel. His directions were that, one catheter being inserted, it was to be extruded from the groove by the withdrawal and reintroduction of the slide, after which the second catheter was to be introduced into the other ureter at the same sitting.

As to proposition 2, that Brown's "composite cystoscope (Fig. 15) involved a wholly novel departure from all preëxisting types," it would seem proper, first, to search Dr. Brown's writings to learn what those features are that are so startlingly original and wholly novel. We read, immediately after the above declaration, that "The first essential change lay in getting rid of the old-time terminal window at the vesical end of the sheath followed, then, by the use of different kinds of interchangeable telescopic tubes for the same common sheath." It would seem that the duty of supplying a wholly novel departure from all preexisting types is hardly fulfilled by getting rid of an old-time terminal window. In reviewing the several sheath instruments that preceded Brown's of 1901, as indicated in the accompanying drawings, it is to be observed that the old-time terminal window appeared in only one, the Brenner,—certainly not a widespread error, to say the least.

We therefore search further for the originality claimed, on which must hinge the wholly novel departure from all pre-ëxisting types; and the next evidence we find of it (ibid) is in the use, as he says, "Of different kinds of telescopic tubes for the same common sheath. Up to the present time its development on this basis has resulted in giving three complete cystoscopes for different purposes, adjustable to one sheath." Verily, Dr. Brown must not have been acquainted with, or must have forgotten, the

Fig. 9.

Schlagintweit

Catheter-cystoscope. (1899)

sheath cystoscopes that had gone before him, or he would never have made such a claim. Boisseau's instrument presented the sheath and multiple-telescope system as plainly as language and artistry could delineate them; in addition to which there were a number of others who made use of the sheath-and-telescope plan, antedating the 1901 instrument of Brown. Preston, of Rochester, in 1898, submitted a sheath and multiple telescope instrument (Fig. 8) to be used with air distention; an instrument that created wide interest and attention througnout this country, and must necessarily have been brought to Dr. Brown's attention.

Other instruments of sheath-and-telescope pattern that antedated Dr. Brown's of 1901 may be mentioned as follows:

1. Güterbock, 1895 (Fig. 4), Berliner Klin Woch., No. 29, 1895.
2. Fenwick, about 1896 (Fig. 5), Clinical Cystoscopy, p. 38.

3. Nitze's Evacuation Cystoscope, 1897 (Fig. 7), Centralbl. f. d. Krankh. der Harn und Sex. Org., 1897, Hft 7, p. 369.
4. Albarran, 1897 (Fig. 6), Revue de gynecologie de Pozzi, No. 3, May and June, 1897.
5. Koch-Preston, 1898 (Fig. 8), Byron Robinson, "History of Cystoscopy," Detroit Med. Journal, October, 1904.
6. Lang, 1899 (Fig. 11), Wiener Med. Presse, 1899, Nos. 27 and 28.
7. Schlagintweit's Evac. Cys., 1899 (Fig. 9), Centralbl. f. d. Krankh. der Harn und Sex. Org., 1900, p. 130.
8. Kollmann, 1900 (Fig. 12), Centralbl. f. d. Krankh. der Harn und Sex. Org., 1900, p. 472.
9. Wossidlo, 1900 (Fig. 14), Centralbl. f. d. Krankh. der Harn und Sex. Org., 1900, p. 461.
10. Bransford Lewis, 1900 (Fig. 13), Jour. of Cutaneous and Genito-urinary Diseases, 1900, p. 420.
11. Tilden Brown's composite, 1901 (Fig. 15), Medical and Surgical Report of Bellevue Hospital, New York, Vol. I., Jan., 1905; Medical and Surg. Report of Presbyterian Hospital, Jan., 1902.

It will be observed that all of these instruments follow after the sheath-and-telescope plan of Boisseau, with individual variations as to details of construction or assembling, and adaptation to different individual objects. This unavoidably close relationship between cystoscopes was recognised and conceded by me in making my presentation, in 1906, before the American Urological Association at Boston, of my universal cystoscope (Fig. 16). Instead of making any claim for originality, the opening paragraph[1] of that introduction was a definite disclaimer in that respect. It says:

In presenting the next candidate for cystoscopic favor, I do so with the full realisation that there is no yawning vacancy in the cystoscopic field; that "there are others" already in the field; also that it is difficult to find anything really new and original, at this date, in the cystoscopic world; that most of the supposed "new" features are simply modified settings or assemblings of features already made use of. It is not desired to lay any especial claim to priority or originality in this instrument; utility is its basic principle.

My idea was that if the instrument as submitted should be found useful, it was at the disposal of the profession; if not, it could easily be retired into innocuous desuetude. But the point of importance here is that in view of the intimate and involved relationship existing between the many cystoscopes on the market, no claim to exclusive individuality or originality could be maintained by any except by Nitze for the fixed-lens system, and by Boisseau for the sheath-and-telescope system. All of the other cystoscopes are but modifications of these two, some good, some bad, the survival of the fittest no doubt best determining the advantage between them.

---

1. American Jour. of Urology, December, 1906.

It remains for us, therefore, to make still further efforts at discovering the features of Dr. Brown's composite cystoscope, that involved a wholly novel departure from all the preëxisting types. I confess my inability to discover a single one. The irrigating features were supplied in several different ways by the sheath instruments depicted in the accompanying drawings,—

Fig. 10. Tilden-Brown-1899.

Fig. 11.

Long (1899)

some with cocks, some with inner tubes for irrigating channels. The differing modes of making the connection with the electric cords are, of course, of little moment, and would not serve as an indicator of particular individuality in a cystoscope. Dr. Brown at first conducted his catheters in closed channels, applied to the lower part of the straight-view telescope (Fig. 10); later, whether receiving his inspiration from Bayard Clark, as he says, or from Follen Cabot, who proposed the change in 1904, he adopted the mode of partially fenestrating the catheter tubes, leaving them open until a bridge is reached at the distal end of the telescope. This differed from modes adopted by others for carrying the catheters, but the difference was hardly sufficient to stamp it as a wholly novel departure from all the preëxisting types. Besides, whose was the credit for the plan finally settled on, and later pictured in Brown's illustrations? (Fig. 15). Not his, by his own assertion.

If, in the extremity of our endeavor to discover the hall-mark of originality, the identity of the "wholly novel departure," we are forced to the brink and name it as the peculiar mode of

Fig. 12. Kollmann – 1900.

applying the lamp to the sheath,—having the lamp constitute the tip of the beak, as used by Brown, 1901,—we are again doomed to disappointment, because Casper[1] and others tell us that exactly that arrangement was applied, in 1880, by Dittel to Nitze's early cystoscopes (Fig. 17); and the Nitze-Dittel cystoscope of 1887, exhibiting that arrangement of illumination, was the outcome. This is one of the classic illustrations used in the earlier writings on cystoscopic history. The lamp formed the tip of the beak; and, as Casper says, this arrangement served well to assist in eliminating the necessity of a cooling apparatus required in connection with the previous plan.

With these facts of cystoscopic history recalled and acknowledged, as answering the first two propositions, the third, I take it, falls of its own weight. In assembling my universal cystoscope I was not reduced to the necessity of appropriating Dr. Brown's composite cystoscope, because there was nothing

1. Casper. Handbuch der Cystoscopie, 1898, p. 16-17.

original in it; like so many others, mine included, it was but a rearrangement of ideas that had already been employed time and again; that have been made use of many times since 1901; and that will be made use of many additional times in the future. The accompanying illustrations of various cystoscopes verify this, —if verification is needed.

One question seems pertinent here—namely, why should I, more than others of the many who have ventured into the field of cystoscopic devising, be so vigorously held to account for having used, in common with Dr. Brown, cystoscopic features that have been handed down from one "originator" to another for a decade? It is true, his big stick has on previous occasions been shaken over the heads of others for their temerity and, having received their chastening, they have retired to dream dreams, but none, so far as I know, have had the honor of such assiduous and pointed attention as I.

However that may be, I am willing to answer the third proposition on its merits and describe wherein my universal cystoscope

Fig. 13. Bransford Lewis Air Cystoscope of 1906 differs from Brown's of 1901, sufficiently to warrant the name it has received. The illustrations themselves (Fig. 15 and Fig. 16) refute the absurd claim that Brown's composite was "the identical instrument which a St. Louis cystoscopist had recently appropriated and was presenting as his universal cystoscope." The following are distinctive differences, as compared with Brown's composite:

First, double fenestration of the beak, instead of the lamp composing the beak. This not only affords better protection to the lamp and its glass chamber, but permits the setting of the lamp upside-down, so to speak. Instead of diffusing its light upward and away from the areas most desired for inspection, as in Brown's (or rather, Dittel's) arrangement, the reversal of the lamp sheds the light most intensely below, on the trigonal area embracing the ureters, bas fond, and other parts most frequently affected with pathological processes; above, toward the prostate and anterior vesical neck, likewise important.

This arrangement of the lamp also permitted the use of a shorter catheterising telescope, which in Brown's catheterising telescope projected a half or three-quarters of an inch beyond the heel of the beak, in a very awkward way, often engaging the lens against the mucous membrane, causing a smear on its surface, and otherwise inviting objectionable results (Fig. 16). One very important one was, that the novice who forgot to withdraw this telescope into the sheath before withdrawing the sheath from the bladder, at once involved himself in trouble and his patient in possible harm. It was like extracting a deep uretral dilator without first closing it. At any rate, whatever the reasons or preferences on the subject, these were essential differences between our instruments.

It would seem that Dr. Brown has come to realise the large advantages incident to this setting of the lamp and the double

Fig. 14. Wossidlo-1900

fenestration of the beak, because in 1907 and again in 1908 while in New York I saw instruments on sale under his name, at his instrument makers, with exactly this arrangement, the lamp having abandoned the tip and being snugly ensconced in the beak itself, set upside down as in mine. If the two sheaths now appear identical, they have become so since the introduction of mine, in 1906, and without any change of form in mine since that time; the inference therefore is plain.

With reference to the direct catheterising telescope, instead of the enclosed catheter tubes at first used by Brown, or the fenestrated which he accepted from either Clark or Cabot, I made use of grooves open throughout but separated by a ridge. This added the space thus given to the telescope and catheter space, the sheath itself completing the walls opposite the grooves.

A more important advantage over the Cabot or Clark bridge arrangement than this was met in practice, when in manipulating a catheter it was withdrawn from under the bridge. Instead of readily and invariably finding its way back into the channel, then pushed forward, it would often become engaged against the bridge, finally requiring the withdrawal of the whole telescope with its catheters to place them again under the bridge. I have seen this happen in the hands of experts with the instrument, so that it is not merely a theoretical objection. In my double grooved channels, no obstacle presents to the progress of the catheters at any point after they are introduced into the channels.

Fig. 15.

With reference to the other telescopes, I have insisted that sufficient space be preserved between the telescope and sheath wall to permit of free exchange of fluid during the work of the operator, whether that be effort at catheterisation or universal inspection with the several telescopes.

In his 1905 reprint, previously mentioned, page 370, Brown says of his composite pattern: "In this the sheath is cylindrical and the examining telescopes fit its lumen snugly, but the catheterising telescope has here to be reduced to a size less than the diameter of the two catheter canals." It is obvious that the snug filling of the sheath by the telescopes leaves no room for the passage of fluid between the two, and that a factor of much value during manipulation is lost thereby.

The retrospective telescope that is common to both instruments has apparently been the occasion of much heat and worriment to Dr. Brown. Whether Dr. Brown or I first suggested the adaptation of the globular lens to a telescope to secure the retrospective view, I know not and care little. I have never made any particular effort to substantiate a claim on the subject, and do not wish to do so now; but I submit the following extract from a letter written under date of June 14, 1904, by the Wappler Electric Controller Company, who made both of our instruments, and should be in position to know the facts: "Dr. Brown and Dr. Cabot are laying the greatest stress on the catheterising feature, and we think the credit belongs to you (Lewis) of suggesting an instrument with the highest grade of retrovision. This we tell every physician who wants to know it. As an examining and irrigating instrument, pure and simple, we can produce one

Fig. 16. Bransford Lewis Universal Cystoscope—1906.

in which your name can be sustained, and we propose to advertise it as the Bransford Lewis Examining Cystoscope."

My first published reference to this retrograde telescope was in 1904, in the *Saint Louis Medical Review*, of December 24, in "Report of a case of hypertrophied prostate." If Dr. Brown published the use of that telescope prior to that time I am not aware of it, and should be glad to have him inform me on the point.

But whether he did or did not, would not invalidate the individuality of my universal cystoscope. Without originality being proclaimed for it, this instrument had at least as much to distinguish it from others as Dr. Brown's instrument, for which claims for unique originality have repeatedly been made,—even to the extent of declaring that it "involved a wholly novel departure from all the preëxisting types."

One more feature connected with my universal cystoscope requires explanation, that is, with reference to the indirect catheterising telescope. Directions and specifications for the making of this feature to be used in the same universal sheath, were given to the Wappler Electric Controller Company in January, 1907. The comparatively simple mechanical difficulties connected with the feature have been overcome in a number of other instruments, both of the direct and indirect catheterising plan, for the past several years,—notably Freudenberg's, Wossidlo's, Casper with Schifka's modification, etc; so that there was no reason to expect undue delay in the production of the feature. Working models were evolved and submitted to me, some being found acceptable, with slight corrections; and so confident was Mr. Wappler of his ability to bring it to prompt completion that the following note was written me on the subject, under date of

Fig. 17. Nitze-Dittel 1887

Lamp at tip of beak

April 2, 1907: "Replying to your favor of 30th ult., beg to state we can furnish the new sheath with indirect catheterising arrangement in about two weeks." Signed, Wappler Electric Controller Co.

What motives or influences have prevailed to prevent the production of this feature of my instrument I can not say; but the unreasonable and inconceivable delay finally resulted in a severance of our relationship and my authorisation of the Kny-Scheerer Company to make the instrument. This tempest in the cystoscopic tea-pot has occurred since that time.

From the above considerations it would seem that the relative positions of Dr. Brown and myself are, that he has been claiming originality and priority that were never due him; and has, after two years, suddenly waxed indignant, and now reproaches me for having used, in accordance with my own ideas, individual cystoscopic features that were present in his instruments and also antedated his from three to ten years. Finally, that the introduction of my cystoscope was unaccompanied by any proclamation of originality, but was submitted wholly and only on the basis of utility.

1050 Century Building.

## Figures and Tables

**Fig. 1. f1:**
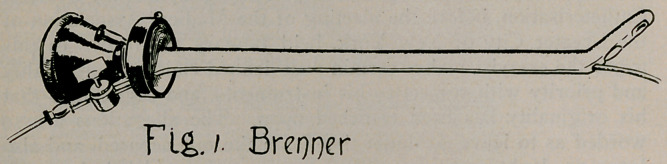


**Fig. 2. f2:**
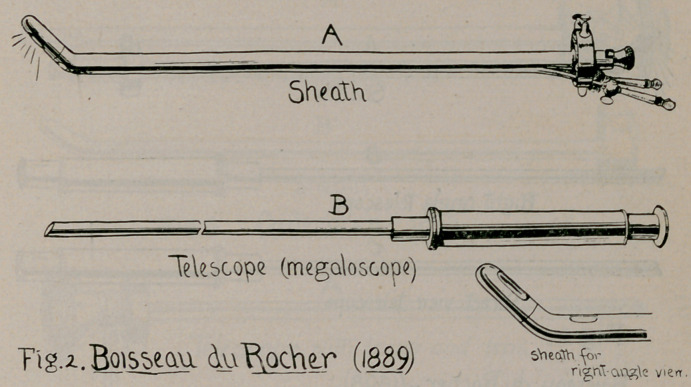


**Fig. 3. f3:**
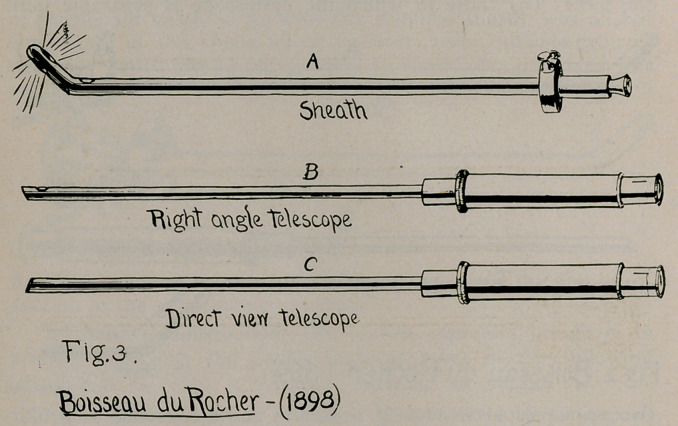


**Fig. 4. f4:**
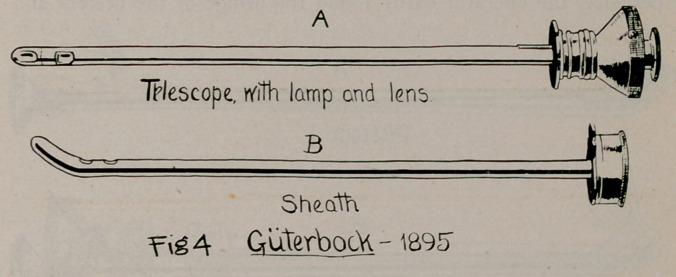


**Fig. 5. f5:**
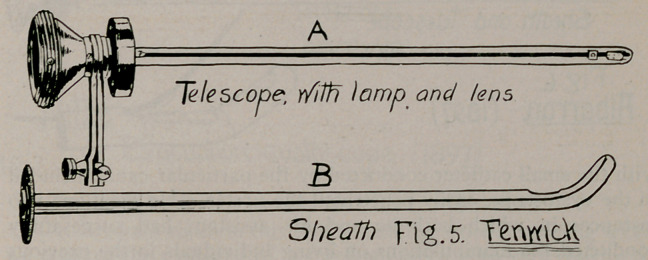


**Fig. 6. f6:**
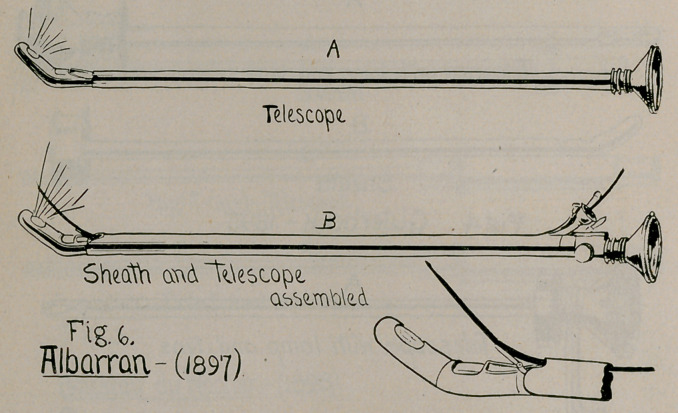


**Fig. 7. f7:**
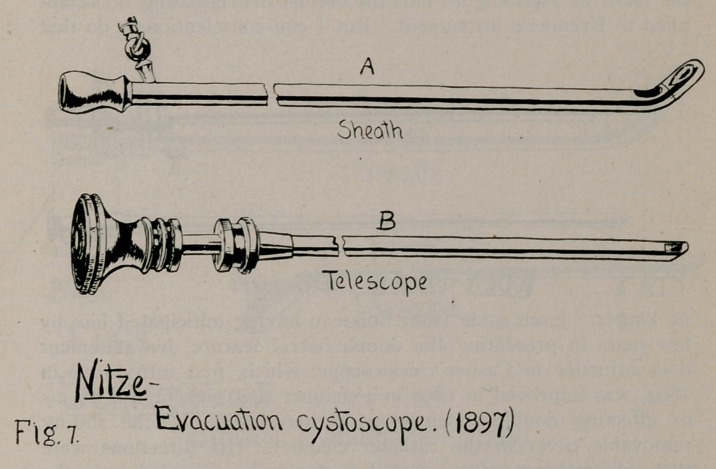


**Fig. 8. f8:**
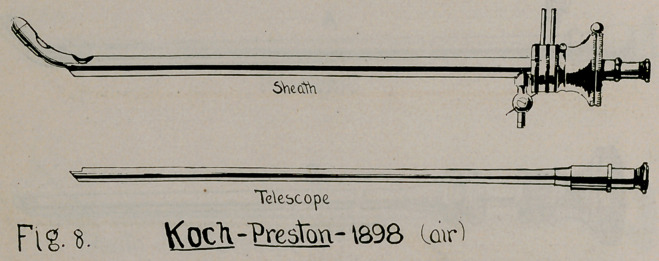


**Fig. 9. f9:**
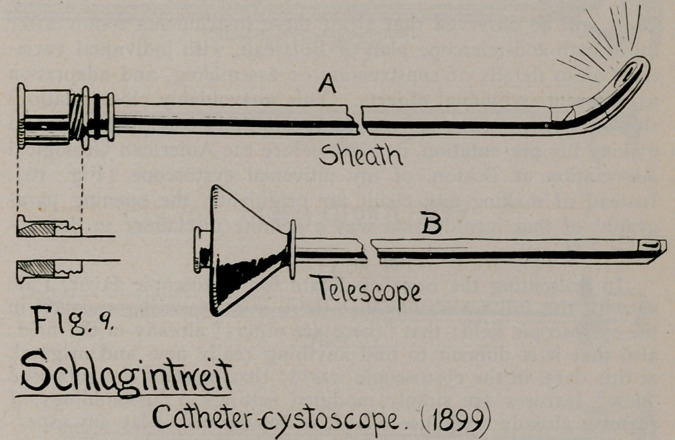


**Fig. 10. f10:**
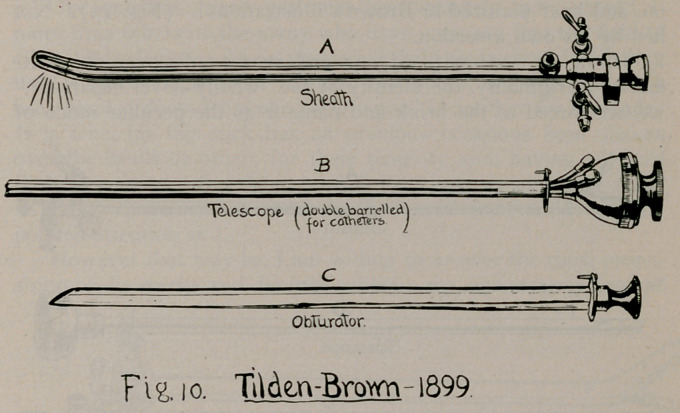


**Fig. 11. f11:**
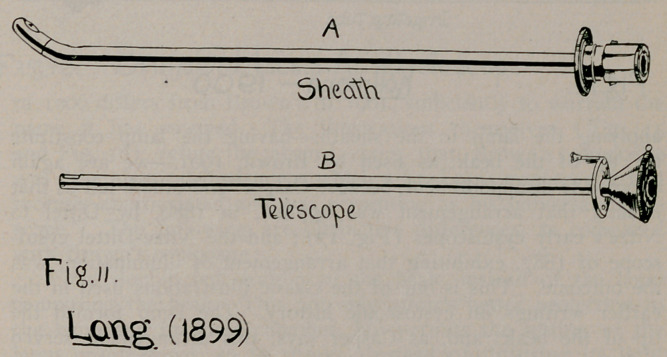


**Fig. 12. f12:**
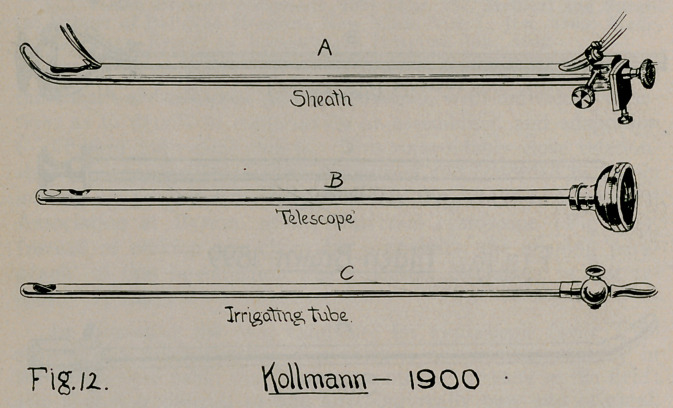


**Fig. 13. f13:**
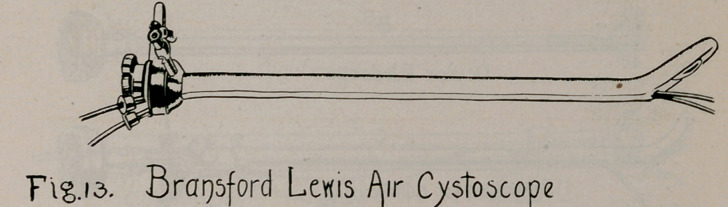


**Fig. 14. f14:**
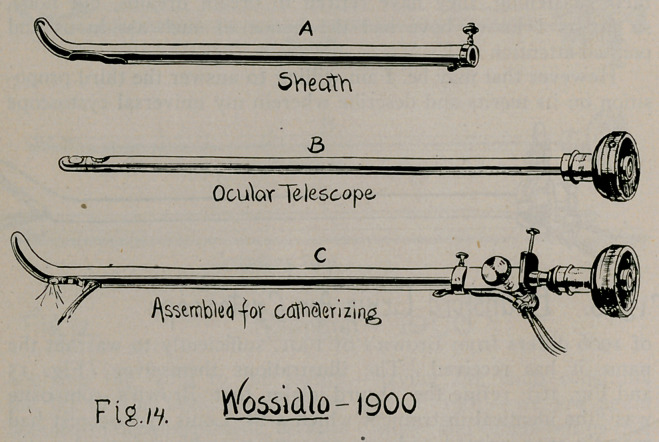


**Fig. 15. f15:**
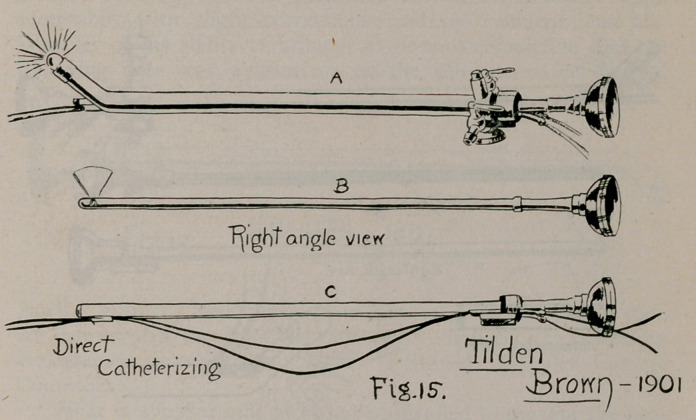


**Fig. 16. f16:**
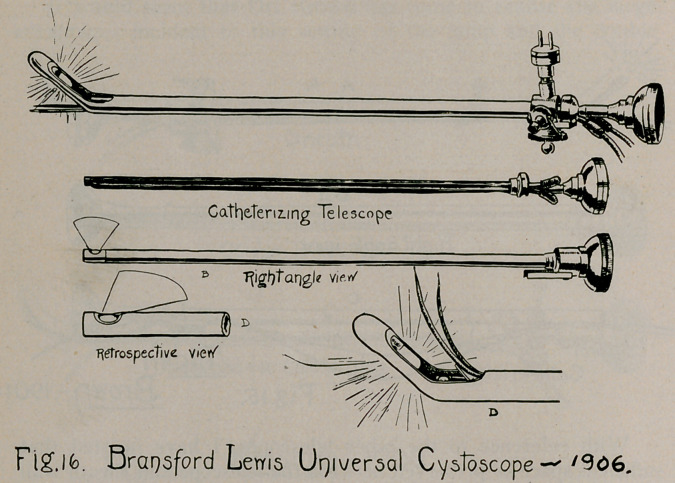


**Fig. 17. f17:**